# Depth-to-scalp spatiotemporal dynamics for stereo-EEG

**DOI:** 10.1016/j.ebr.2025.100784

**Published:** 2025-06-13

**Authors:** Tal Benoliel, Oshrit Arviv, Diya Doufish, Netaniel Rein, Yuval Harpaz, Evgeny Tsizin, Michal Balberg, Sami Heymann, Zvi Israel, Mordekhay Medvedovsky, Dana Ekstein

**Affiliations:** a Department of Neurology and Agnes Ginges Center for Human Neurogenetics, Hadassah Medical Organization, Hadassah Ein Kerem, POB12000 Jerusalem, Israel; b The Faculty of Medicine, Hebrew University of Jerusalem, Hadassah Ein Kerem, POB12000 Jerusalem, Israel; c Inner Eye Ltd, Israel; d Holon Institute of Technology, POB 305, Holon 5810201, Israel; e Department of Neurosurgery, Hadassah Medical Organization, Hadassah Ein Kerem, POB12000 Jerusalem, Israel

**Keywords:** SEEG, Epilepsy, Epilepsy surgery, Interictal epileptiform activity

## Abstract

The data obtained from stereo-elecroencephalography (SEEG) in patients with focal epilepsy are crucial for defining the epileptogenic zone and achieving successful resection, but suboptimal electrode placement impairs SEEG results. We demonstrate an approach for concurrent scalp and depth EEG analysis from one patient with successful intracranial workup and one in whom the seizure onset zone was unsampled by SEEG. Intracranial epileptiform discharges were identified and clustered, their scalp correlates were averaged, and electric source imaging (ESI) was applied to the resulting averaged scalp potential – depth-to-scalp ESI (dsESI). We found temporal differences between intracranial and scalp peaks, as well as variations in averaged scalp spikes morphology and propagation, expressed by their amplitudes and width, and by their jitter across involved electrodes. Put together with the relative degree of focality and location of the averaged scalp spikes’ ESI on the cortex, these data could differentiate onset from propagation of interictal activity and identify unexplored nodes in the epileptic network. Our novel analysis highlights the importance of temporal, and not just spatial, spike dynamics within the epileptic network, may be used to validate depth electrode placement and aid in understanding the epileptic network.

## Highlights

• Averaging intracranial epileptiform discharges unmasks obscured scalp potentials.
• Averaged scalp fields’ and depth spikes’ temporal interplay informs spike spread.
• Electric source imaging of averaged scalp potentials uncovers network nodes.
• This analysis can be used to validate SEEG coverage.

## 1 Introduction

In focal-onset drug resistant epilepsy (DRE), stereo-electroencephalography (SEEG) is instrumental in defining the surgical target [1,2]. However, SEEG's high spatial resolution is limited by patchy spatial sampling, contributing to epilepsy surgery failures [3,4].

Scalp EEG has wide spatial sampling but reflects the synchronous activity of large cortical areas [5]. It is frequently added to clinical SEEG studies to ascertain that intracranial ictal onset is not preceded by scalp EEG. Averaging of scalp activity time-locked to intracranial interictal epileptiform discharges (iIEDs) was shown to reveal unseen scalp correlates [6,7], and ESI of scalp EEG potentials during iIEDs [7] and intracranial electrical stimulation has been used to test the accuracy of source localization methods [8,9]. In addition, the temporal propagation of spikes, spike onset identification and resection of spike onset regions has been linked to improved postsurgical outcomes [10].

In this study, using two example cases, we illustrate a novel approach to explore the spatiotemporal dynamic of iIEDs with concurrent scalp EEG. To this end, electric source imaging (ESI) of iIEDs-locked averaged scalp fields (ASF) was performed-referred to as depth-to-scalp ESI (dsESI). We hypothesized that the temporal relation between scalp and depth spike peaks, and the extracted scalp sources may provide non-redundant information regarding the spatiotemporal characteristics of epileptic networks helping differentiate spike onset from propagation, validate SEEG electrode placement, and potentially identify unexplored network nodes.

## 2 Materials and methods

Two DRE patients undergoing SEEG with concurrent scalp EEG monitoring were included in the study, approved by local IRB with waiver of informed consent. One had successful workup targeting temporal lobes bilaterally resulting in bitemporal RNS implantation and long-lasting seizure freedom, while in the other patient clinical seizure onset preceded intracranial electrodes involvement, indicating SEEG did not cover the seizure onset zone (SOZ). iIEDs were manually annotated during wakefulness and sleep, and clustered automatically using fuzzy c-means clustering followed by manual clustering validation by a trained epileptologist (TB). Scalp EEG time-locked to iIEDs, aligned by spike peak at highest amplitude channel, was averaged within clusters. Difference in timing of ASF peak compared to iIED peak were obtained. Inter-channel jitter, indicating the variability in spike timing across channels as a simple metric of propagation, was calculated by taking the mean of the absolute difference in peak timing between involved channels. ASFs were morphologically analyzed, and ESI using sLORETA was performed on ASFs alone [11]. Distributed source estimations were evaluated using a spatial dispersion (SD) metric. For more information, see figure legends and supplementary methods.

## 3 Results

**Fig. 1:**
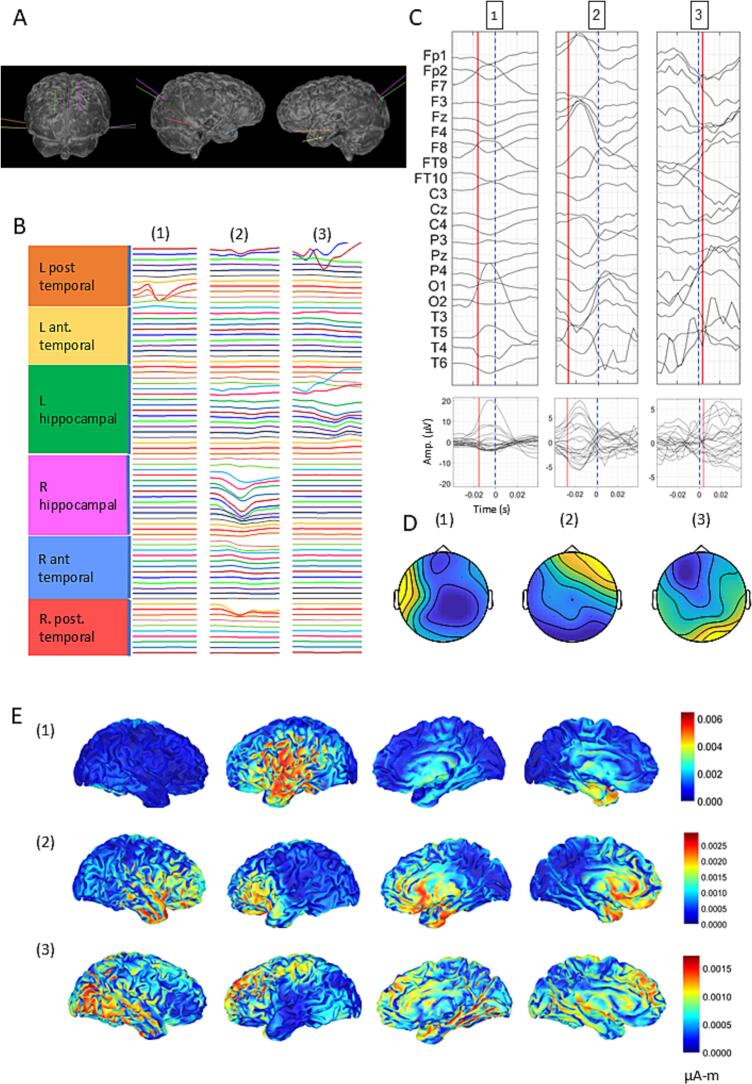
Patient 1 was MRI-negative, investigated with bilateral temporal SEEG (A) that suggested a left basal temporal SOZ rapidly spreading to the hippocampi, and now over 3 years seizure-free with bilateral RNS. Three clusters of averaged iIEDs (B) are shown. The averaged scalp potentials (µV) are shown with time 0 as the intracranial peak, and a vertical red line at mid-peak (C) as well as their respective scalp topographies at mid-peak (µV) (D). Current density maps in 4 different views obtained via sLORETA (µA-m) at mid-peak are shown for each cluster in (E). (For interpretation of the references to colour in this figure legend, the reader is referred to the web version of this article.)

In patient 1, 548 spikes, 522 of which were not recognizable on unaveraged scalp EEG, were allocated into three clusters (Fig. 1A, B). All clusters had a clear ASF (Fig. 1C), though cluster amplitude, width and inter-channel jitter differed (Cluster 1, left basal temporal iIED: 17.6 µV amplitude, 23.4 ms width, 1.22 ms jitter across scalp channels; cluster 2, right hippocampal iIED: 6.8 µ V amplitude, 19.5 width, 0.71 ms jitter; cluster 3, left hippocampal iED: 3 µV amplitude, 43 ms width and 3.25 ms jitter, see Supplementary Table 1). Observing the delta between iIED and ASF peaks, cluster 1 ASF peak preceded iIED peak by only 3.9 ms, cluster 2 ASF peak preceded depth peak by 15.6 ms, implying that right hippocampal activity likely signifies spike propagation, and cluster 3 ASF trailed the iIED peak by 19.5 ms, suggesting left hippocampal origin.

dsESI (Fig. 1D) in Cluster 1 (Fig. 1D(1), Supplementary Video 1a), localized to the left temporal operculum at spike onset (39 ms pre-peak) spreading to temporal pole and mesial temporal lobe (20 ms pre-peak) and to right mesial temporal structures (20 ms post-peak). Sources' SD was low and similar at mid-peak and peak (Supplementary Table 2). Cluster 2 (Fig. 1D(2), Supplementary Video 1b) localized to left opercular, right frontotemporal and bilateral mesial temporal areas at spike onset and throughout the spike time-course, and showed a stable, low SD of sources (Supplementary Table 2). Cluster 3 (Fig. 1D(3), Supplementary Video 1c), showed left frontotemporal, opercular, temporal polar and bilateral mesial temporal involvement at spike onset, spreading to the mesial temporal structures bilaterally at mid-peak and to lateral parietal and temporal regions at peak. SD of sources was higher at mid-peak than peak (70, 96.1 for positive and negative dipoles, respectively) than at peak (57.7, 56.1) and compared to patient's other clusters (from 48.8 to 58.2; Supplementary Table 2).

Collectively, these spatiotemporal patterns imply that spike onset may be in the left temporal regions, perhaps near the left hippocampus, complementing the patient's right and left hippocampal seizures, the latter of which showing left basal temporal onset (Supplementary Fig. 1A–C), though an independent right hippocampal focus cannot be excluded.

**Fig. 2:**
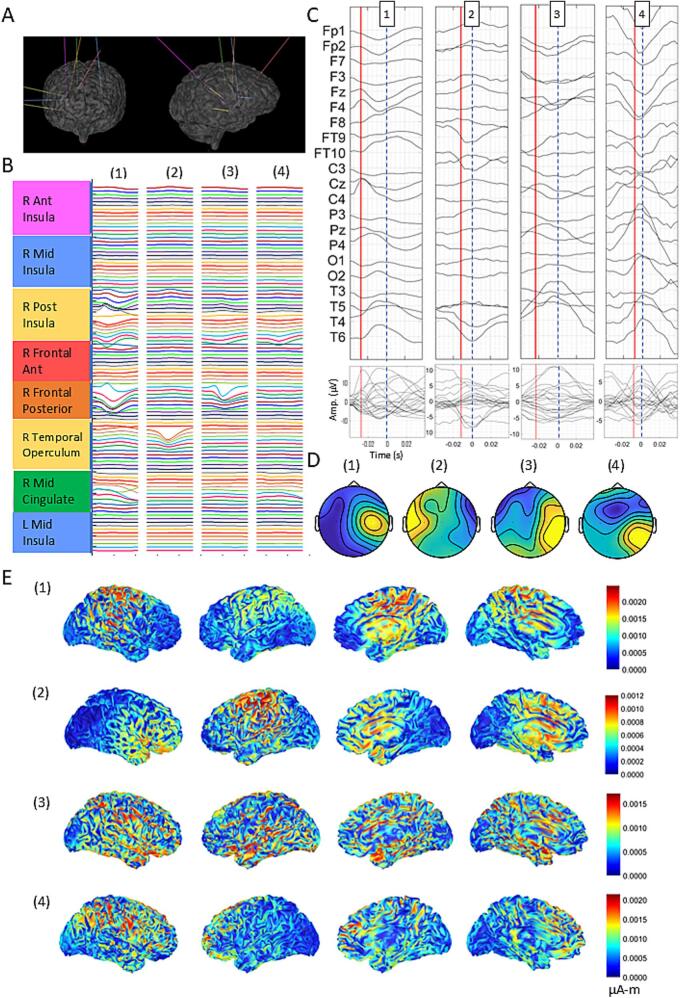
Patient 2 was MRI-negative, investigated with right fronto-perisilvian-insular SEEG (A), where all intracranial ictal activity was preceded by clinical seizure onset, therefore no surgical intervention was advised. Four clusters of averaged iIEDs (B) are shown. The averaged scalp potentials (µV) are shown with time 0 as the intracranial peak, and a vertical red line at mid-peak (C), as well as their respective scalp topographies at mid-peak (µV) (D). Current density maps in 4 different views obtained via sLORETA (µA-m) at mid-peak are shown for each cluster in (E). (For interpretation of the references to colour in this figure legend, the reader is referred to the web version of this article.)

In patient 2, 287 of 439 iIEDs were undetectable on scalp EEG. iIEDs were clustered semiautomatically into four clusters, linked to right peri-Sylvian, insular, and cingulate contacts (Fig. 2A, B, Supplementary Table 1). All iIED-locked ASFs showed irregular scalp potentials (Fig. 2C), with amplitudes of 7.7–15.8 µV, widths of 23.4–54.7 ms, and jitter of 1.95–3.51 ms across channels (Fig. 2C, Supplementary Table 1). ASFs in clusters 2 and 4 peaked simultaneously on the scalp and intracranially, while clusters 1 and 3 peaked 11.7 ms and 7.8 ms before the intracranial spike, respectively.

dsESI for cluster 1 (Fig. 2D(1), Video 2a), from iIEDs in the right posterior insula, anterior cingulate, and posterior frontal areas, showed bilateral temporoparietal scalp sources at mid-peak, quickly spreading widely. Cluster 2, from right temporal operculum iIEDs, displayed left superior frontal and bilateral mesial fronto-temporo-parietal sources at mid-peak, with diffuse cortical spread (Fig. 2D(2), Supplementary Video 2b). Clusters 3 and 4 (Fig. 2D(3–4), Supplementary Videos 2c–d showed no discrete sources at mid-peak, rapidly engaging broad cortical areas at peak. High SD occurred in all clusters (65.3–77.2), except for cluster 1 at mid-peak (positive dipole: 54.4, negative dipole 55.8).

Together, the absence of early iIED peaks, irregular spike morphology, high jitter, and dispersed sources in all clusters suggest suboptimal seizure network sampling in patient 2; notably, clinical seizure onset preceded intracranial recordings (Supplementary Fig. 1D), indicating key nodes were unsampled.

## 4 Discussion

This study demonstrates how combining SEEG and scalp EEG enhances invasive investigations. Our novel approach exploits the abundance and high SNR of iIEDs to reveal concomitant occult scalp EEG potentials and demonstrates the added value of the temporal dynamic between ASF and iIED peaks in addition to spatial aspects.

We used the spatiotemporal relation between the SEEG and scalp peaks to extract spatial spike propagation. In addition, in some cases (Supplementary Video 1a) a clear and contiguous spatial spread of the sources throughout the scalp spike time course delineates regions of early versus late spike involvement. This can both help validate a scalp EEG source and identify spike onset which is valuable in defining the epileptogenic zone [12] as the resection of spike generators [13] as well as sources of early versus late propagating iIEDs [10,14] has been shown to correlate with favorable postsurgical outcome. Scalp and SEEG peak disparities and scalp EEG source evolution may be combined to infer the temporal sequence of spike propagation.

The disparity between ASF and iIED peaks may be used to validate SEEG coverage. While the temporal disparity between scalp and depth peaks might be due to conduction velocity of electrical activity or signal averaging, it can be inferred that when scalp activity precedes intracranial activity, the intracranial finding represents signal propagation. Our analysis suggests that when all scalp correlates precede their intracranial counterparts, the intracranial investigation does not cover the SOZ. However, when iIEDs precede ASF peaks or are synced with them, the results are more difficult to interpret, though the former may imply that interictal activity has not massively propagated at the point of intracranial sampling. Gaining this information from iIEDS, prior to seizure acquisition, may indicate the need to elaborate SEEG coverage. Future studies will be needed to investigate in depth the temporal relation between iIEDs and ASFs and the resulting ESI locations.

In addition to SEEG validation and inferring interictal temporal dynamics, our findings indicate that applying ESI to ASF may identify additional, unsampled regions. This adds to previous work showing that ESI applied to ASF can localize the depth electrode from which iIEDs were recorded [7]. Thus, if depth coverage in case 1 had been right unilateral, left temporal involvement would have been estimated nonetheless, demonstrating our analysis's potential to identify unsampled regions. In patient 2, our analysis failed to localize novel targets, partly due to dispersed sources. This may be due to low SNR, which impedes ESI, a relatively small number of scalp electrodes, a broad or multi-nodal onset in the spike network, or overestimation of superficial sources when interictal activity originates from deep brain regions. Future studies implementing depth and scalp potentials based ESI may overcome the limitations of scalp surface-biased source localization and reduce localization errors [15,16].

This pilot study is limited by a small patient sample; automated spike annotation in a larger, prospective cohort of epilepsy surgery candidates could enhance and validate the analysis.

## 5 Conclusion

iIEDs and their triggered ASFs can be combined to validate SEEG studies and advance clinical understanding of network nodes and spatiotemporal dynamics. With further testing through additional cases, this approach may be incorporated into the SEEG pipeline and used to elaborate depth electrode coverage.

## Ethical statement

All procedures were performed in compliance with relevant laws and institutional guidelines. The study HMO-0216-12 was approved by the institutional IRB at Hadassah Medical Center (Helsinki committee) on 12th August 2012. The committee regularly reviews and monitors the study's progression and has authorized its continuation until 9th August 2025.

The privacy rights of human subjects have been observed, in this study the need for informed consent was waived by the local IRB.

## CRediT authorship contribution statement

**Tal Benoliel:** Writing – original draft, Methodology, Data curation. **Oshrit Arviv:** Writing – review & editing, Writing – original draft, Software, Methodology, Formal analysis. **Diya Doufish:** Writing – review & editing. **Netaniel Rein:** Writing – review & editing. **Yuval Harpaz:** Software. **Evgeny Tsizin:** Writing – review & editing, Conceptualization. **Michal Balberg:** Writing – review & editing. **Sami Heymann:** Data curation. **Zvi Israel:** Writing – review & editing, Data curation. **Mordekhay Medvedovsky:** Writing – review & editing, Supervision, Methodology, Conceptualization. **Dana Ekstein:** Writing – review & editing, Supervision, Conceptualization.

## Declaration of competing interest

The authors declare the following financial interests/personal relationships which may be considered as potential competing interests: Evgeny Tsizin, Michal Balberg and Mordekhay Medvedovsky are inventors in patent WO2023242838A1. None of the other authors has any conflict of interest to disclose.
