# Supplementary material for: Depth-to-scalp spatiotemporal dynamics for stereo-EEG
Source: Epilepsy Behav Rep. 2025 Jun 13;31:100784. doi: 10.1016/j.ebr.2025.100784 (PMC12399257; doi:10.1016/j.ebr.2025.100784)
Supplement: Supplementary Data 8 [file mmc8.docx]

**Supplementary Materials and Methods**

*Patients and Data acquisition*

Patients underwent implantation with depth electrodes (DIXI 12-15 contacts, interval 3.5 mm, diameter 0.8mm). The location, trajectory and number of electrodes was determined based on the presurgical evaluation. In addition, scalp EEG, using 21 electrodes placed in accordance with the 20-10 system, was acquired. Data were collected using the XLtec clinical system (Natus Medical Inc.), sampled at 2048 HZ and downsampled to 256 Hz. MR images (voxel size: 1 mm^3^) were obtained from a 3T MRI scanner (Phillips). The clinical information of the patients is summarized in a separate section.

Data analysis

Data processing and analysis were performed using MATLAB 2020a (The MathWorks) and Brainstorm[1]. Intracranial interictal epileptiform discharges (iIEDs) were identified on SEEG recordings by an experienced epileptologist (TB), and their first peak was marked.

The locations of the electrodes were determined by projecting to each patient’s brain MRI images and anatomical landmarks: ear, nasion and inion, to decrease the mismatch between true electrode locations and those used in analysis.

1. *Spike clustering*

SEEG spikes were clustered using a semi-automatic procedure, as following: First, measured signals were threshold (signal duration: approximately 80 msec (± 10 samples) surrounding the peak of each discharge, threshold: ±2.5 standard deviations). Subsequently, each entry of a matrix (spike, depth electrode) was assigned with the number of crossings along with the sign as the majority of crossings’ polarities at the specific depth electrode contact. Accordingly, information regarding spatial distribution and directionality of the epileptiform activity was sparsely maintained. Fuzzy c-means clustering was applied with number of clusters set to the elbow point of minimal intra-distances between clusters as a function of the number of clusters. The results of this coarse sorting were reviewed by a trained epileptologist (TB). In patient 1, only small modifications were made to the suggested classification, while in patient 2, the suggested 2 clusters were manually further divided and modified into 4 topographically distinct spike populations. Mainly, one of the clusters appeared to contain spikes of diverse spatial distributions and thus, was manually divided into three groups, based on spike topographic distribution and morphology. Additionally, the concurrent scalp EEG traces were scrutinized to determine whether (or not) epileptiform activity was visualized on the scalp at the time of the intracranial spike, and iIEDs were divided into two groups accordingly.

1. *source estimation and evaluation*

Scalp EEG time locked to intracranial spikes was averaged across clusters to reveal the scalp component of intracranial epileptiform activity. Source localization was implemented on the averaged scalp fields, using a common technique, standardized low-resolution tomographic analysis (sLORETA)[2]. sLORETA offers a distributed inverse solution, estimating the amplitude of sources, represented by a grid of current dipoles distributed across the brain, and therefore does not require specifying a priori the number of sources. The obtained solution, a current density map, was computed using the following: the boundary element method (BEM) was used to compute the gain matrix based on each patient’s MRI and the open-source software OpenMEEG[3, 4], the grid of current dipole sources was limited to the cortical surface, and the orientations of these current dipoles were constrained to the normal direction of the cortical surface, regularization parameter corresponding to a signal to noise ratio equal to three, an estimate of the noise covariance was generated from a baseline activity before discharges of epileptiform events.

There is an ongoing controversy as to what instance in a spike trace should be used for source analysis[5, 6]. In this study all sampled timepoints surrounding the peak of the averaged discharges (~80 msec, ± 10 samples) were analyzed. For display purposes, only the mid peak was presented. The mid phase of a discharge is considered a compromise between the higher signal-to noise ratio (SNR) of the peak and the reduced potential of cortical-cortical propagations, away from the original cortical generator of the discharge in its onset phase. The mid-peak was defined as the timepoint of 50% mean global field power (GFP) over the baseline.

To identify the sources providing the strongest solutions for each scalp potential, two dipoles showing most positive, most negative, or one most positive and one most negative intensity were considered [7]. The sign indicates the polarity of the source relative to the cortex's normal surface. Artificially projected scalp potentials from each of these three couples of dipoles were compared to the original scalp pattern, using a normalized relative difference measure[8], and the option that better explained the scalp EEG pattern was chosen and ascribed anatomical labels according to the Desikan-Killiany atlas[9]. To quantify the source distribution density comparative to the specified dipoles, a spatial dispersion (SD) metric was used [7], calculating the squared root of the relation between the sum of intensities (s_i_ marks each intensity) weighted by the distance from a selected dipole (D_ij_ marks the distance between dipole i to strongest dipole j) to the sum of intensities, so that a larger spatial dispersion score reflects solutions more diffuse relative to the particular strongest dipole of interest.

$SD= \sqrt{\frac{\sum_{all dipoles} ({min}_{j=strongest}D_{ij}^{2})s_{i}^{2}}{\sum_{all dipoles} s_{i}^{2}}}$ (1)

**Supplementary Clinical Background**

*Patient 1*

A healthy 27-year-old female developed DRE, with seizures featuring anxiety, a floating sensation, and impaired concentration, progressing to mastication, unresponsiveness, and tonic-clonic episodes. MRI was normal, video-EEG monitoring revealed bilateral independent temporal ictal activity and interictal epileptiform discharges, and PET-CT indicated left temporal hypometabolism. Invasive monitoring targeting the mesial and lateral temporal lobes bilaterally suggested seizures originating from the left inferior temporal region and spreading to the left hippocampus. Although a left temporal neocortical epileptogenic zone was likely, the presence of a second right temporal focus couldn't be ruled out. Treatment options were weighed between left temporal resection and bitemporal closed-loop stimulation using Responsive Neurostimulation (RNS). Opting for the latter, electrodes were implanted, resulting in seizure freedom for 24 months without recorded stimulations, despite no changes to her antiseizure medications (ASMs).

*Patient 2*

A 20-year-old female had DRE since the age of three years, hypothyroidism since the age of 15, and a positive family history of epilepsy in two siblings. Her seizures presented with right unilateral blinking and left facial twitching, followed by right gaze deviation and hyperkinetic seizures, which occurred several times per day. MRI was non-lesional, and VEM showed right frontal IEDs and right frontal seizures. The onset was presumed to be right frontal and a SEEG investigation targeted the right posterior frontal cortex, temporal operculum, insula and anterior parietal lobe.  Seizures began clinically before they appeared electrographically on SEEG, and initially involved depth electrodes in the posterior insula and temporal operculum. Given the electroclinical delay seen in the ictal recordings of the SEEG investigation, we concluded that the epileptogenic zone was unsampled by the depth electrodes.

|  | Cluster no. | No. of spikes | No. of spikes – scalp display | Amplitude  (µV) | Width  (msec) | Peak time*  (msec) | Intracranial regions involved | No. (identity) of scalp channels involved** | Inter-Channel Jitter*** (msec) |
| --- | --- | --- | --- | --- | --- | --- | --- | --- | --- |
|  |  |  |  | (at maximal \|channel\|) | |  |  |  |  |
| Patient 1 | 1 | 297 | 12 | 17.6 | 23.4 | -3.9 | Left subtemporal neocortical | 16 (FP1, FP2, F7, F3, Fz, F4, F8, T3, C3, Cz, C4, T5, Pz, P4, T6, FT9) | 1.22 |
|  | 2 | 118 | 9 | 6.8 | 19.5 | -15.6 | Right hippocampus | 11 (FP2, Fz, F4, F8, C3, T5, P3, Pz, O1, O2, FT10) | 0.71 |
|  | 3 | 133 | 7 | 3.0 | 43.0 | 19.5 | Left hippocampus | 6 (FP1, F3, C3, T6, O2, FT10) | 3.25 |
|  |  | Total: 548 | Total: 28 |  |  |  |  |  |  |
| Patient 2 | 1 | 155 | 126 | 15.8 | 39.1 | -11.7 | Right posterior insula, anterior cingulate and posterior frontal | 10 (Fp1, Fp2, F3, Fz, C3, T4, P3, P4, T6, O2) | 3.51 |
|  | 2 | 72 | 6 | -7.7 | 31.3 | 0.0 | Right temporal operculum | 8 (Fp1, Fp2, Fz, F8, Cz, T4, P3, FT10) | 1.95 |
|  | 3 | 177 | 15 | 11.15 | 54.7 | -7.8 | Right posterior frontal | 5 (Fz, Cz, T4, P4, O2) | 3.12 |
|  | 4 | 35 | 8 | 9.1 | 23.4 | 0.0 | Posterior insula and cingulate | 6 (F3, Fz, C4, T4, Pz, P4) | 3.25 |
|  |  | Total: 439 | Total: 155 |  |  |  |  |  |  |

Supplementary table 1: Clusters and averaged scalp spikes

* Relative to intracranial

** Above/below mean of baseline ± 2SEM

*** The inter-channel jitter was calculated as the mean of |∆T_peak_| over channels.

Supplementary table 2: Source metrics

|  | Cluster no. | Quantitative Mid-peak sources* | Mid-peak Spatial dispersion | Peak sources* | Peak Spatial dispersion | Qualitative Mid-peak sources** | ESI sampled by SEEG electrodes? | conclusion |
| --- | --- | --- | --- | --- | --- | --- | --- | --- |
| Patient 1 | 1 | Left parsopercularis | 54.5  56.6 | Left superiortemporal | 48.8  51.6 | Lt temp operculum, temp pole | yes | ESI traced back SEEG location |
|  | 2 | Right medialorbitofrontal | 55.7  57.1 | Right medialorbitofrontal | 56.6  58.2 | Rt + Lt mesial temporal | yes | ESI uncovered left sources, which were not active though sampled. (Generator probably near) |
|  | 3 | Right pericalcarine  Left rostral middle frontal | 70.0  96.1 | Right lingual  Right precuneus | 56.7  55.1 | Lt  hippocampus  Rt posterior temporal | yes | ESI traced back SEEG location and additional unsampled posterior temporal region (propagation) |
| Patient 2 | 1 | Right paracentral  Right precentral | 54.4  55.8 | Right superior temporal  Right transverse temporal | 69.5  67.5 | Rt superior frontal | yes | ESI traced back SEEG location |
|  | 2 | Left postcentral | 70.8  73.3 | Right insula  Right superior temporal | 67.9  74.4 | Lt superior frontal | yes | ESI pointed to distal location, where no concurrent activity was recorded (sources too dispersed) |
|  | 3 | Left fusiform  Left superiorparietal | 77.2  71.8 | Left lingual  Right postcentral | 65.3  66.1 | Too dispersed | - | - |
|  | 4 | Right postcentral | 69.0  67.6 | Right postcentral  Right precentral | 67.9 | Too dispersed | - | - |

* Please see ‘source estimation and evaluation’ section above

** A qualitative description of source density maps' distribution

**Supplementary Figure 1**

(A)


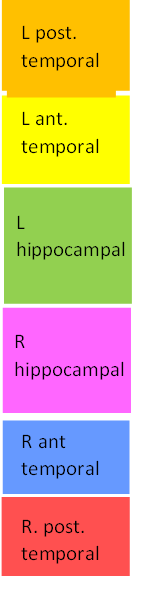

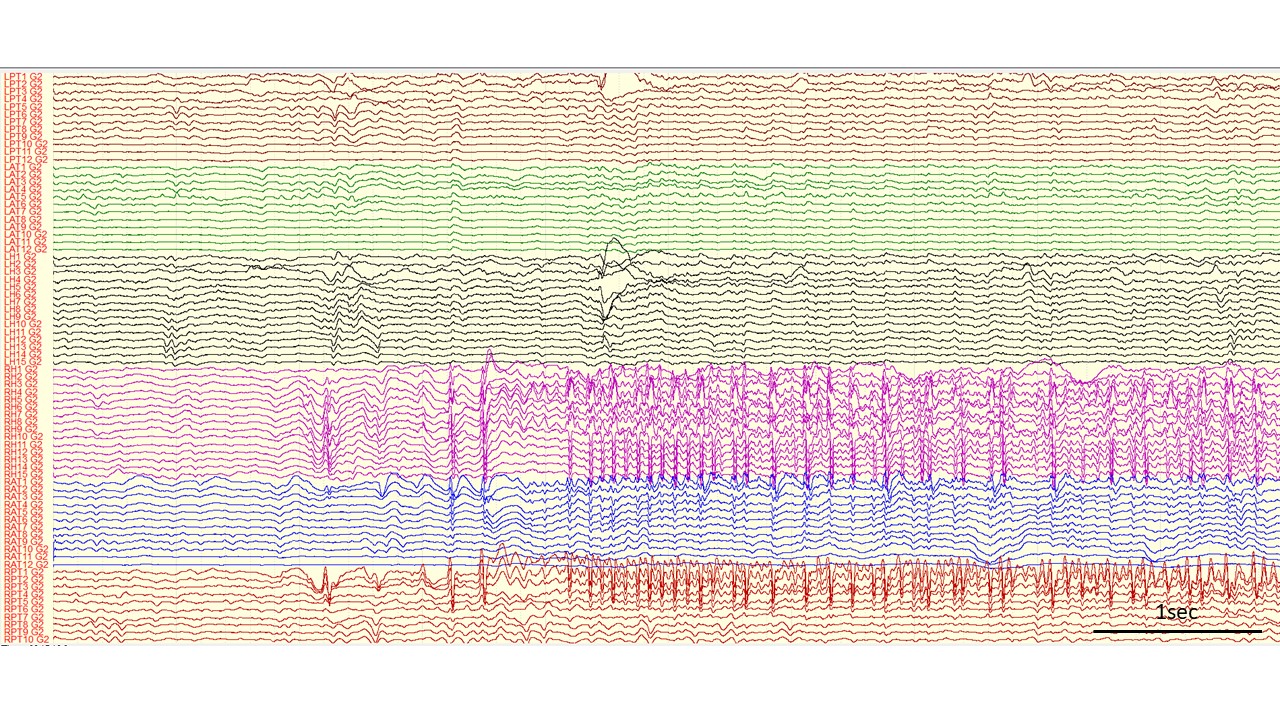
(B)


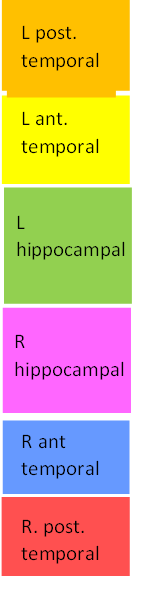

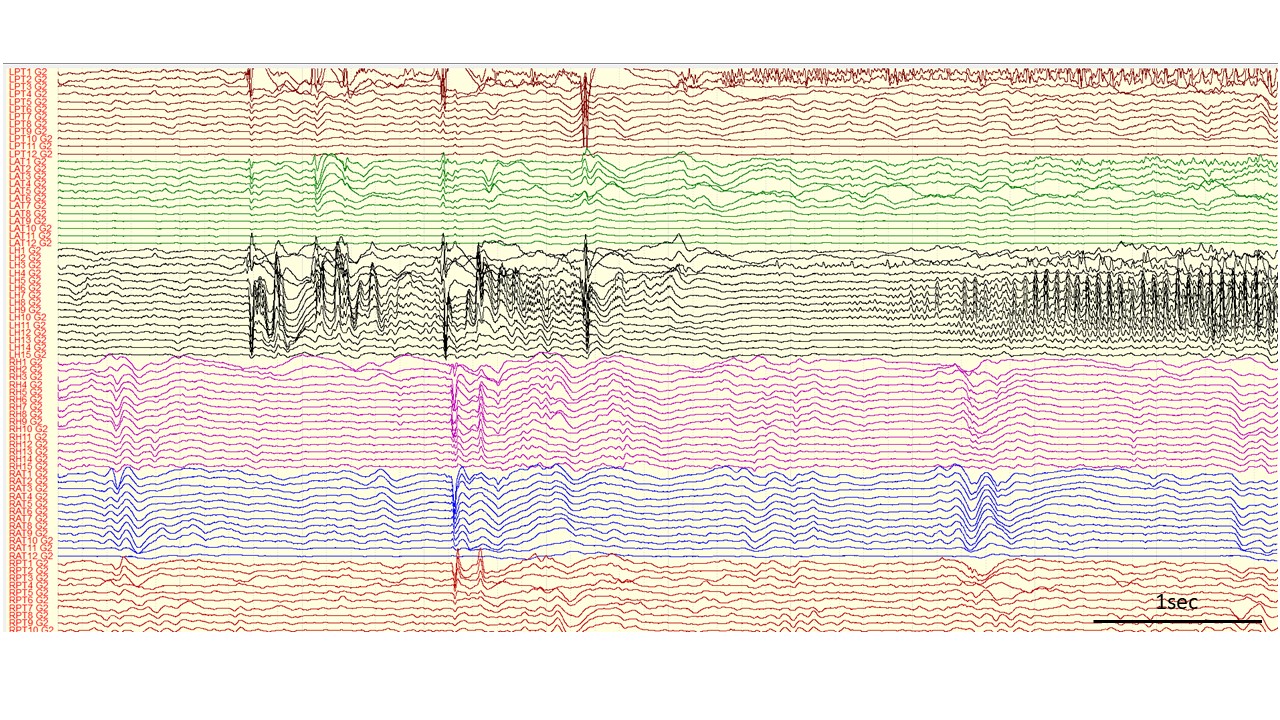


(C)


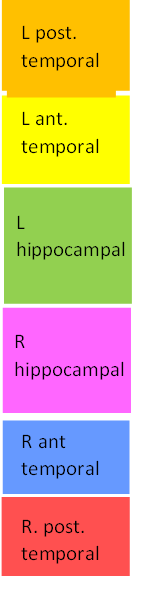

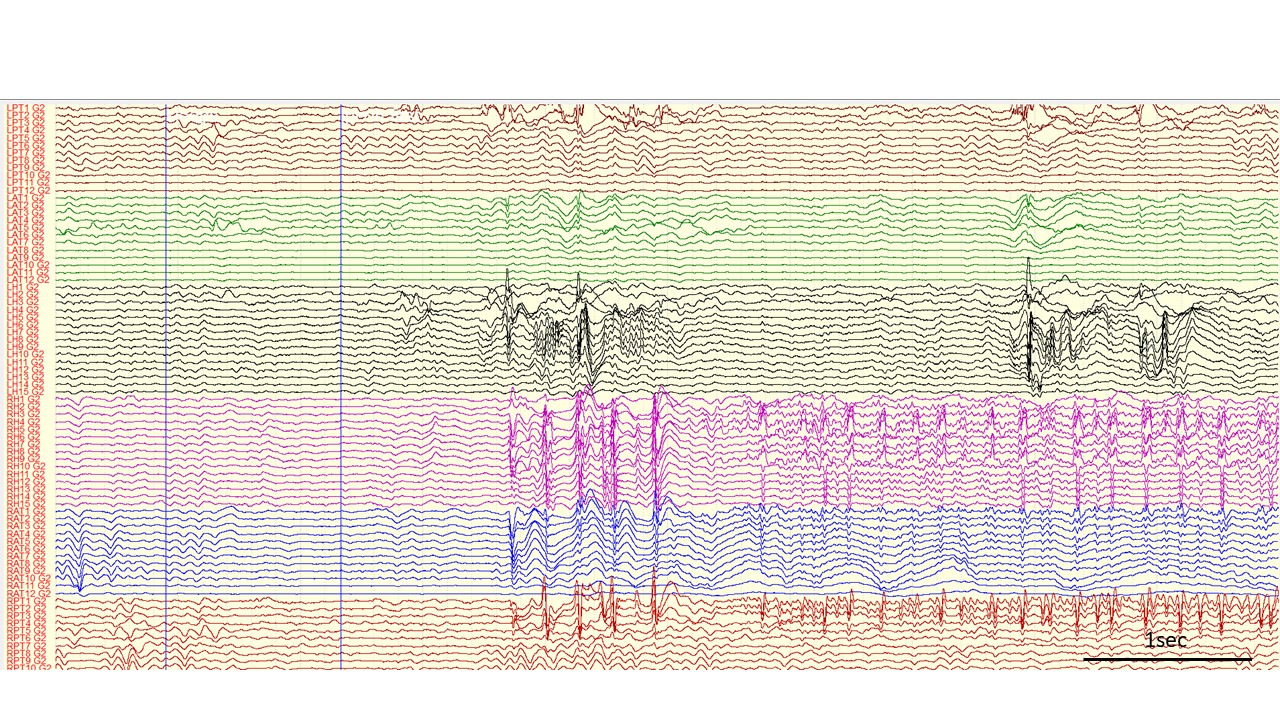


(D)


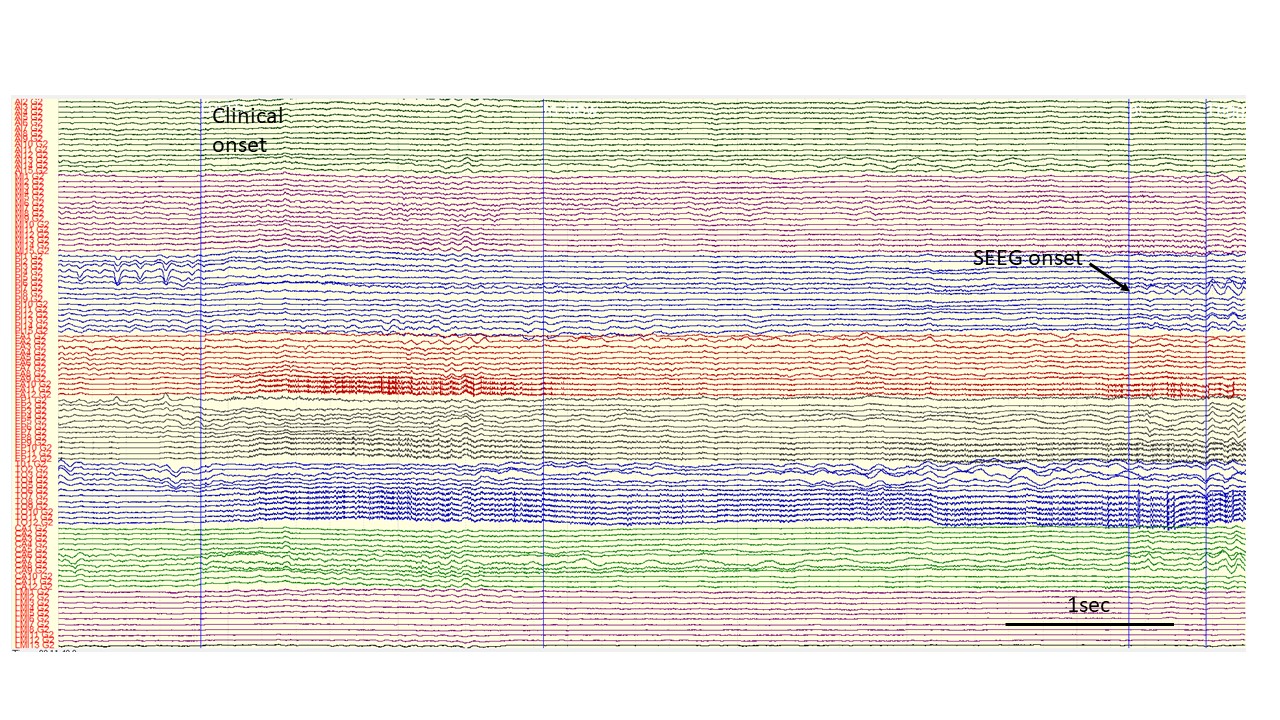


R Ant Insula

R Post Insula

R Mid Insula

R Frontal Ant

R Frontal Posterior

R Temp. Operculum

R Mid Cingulate

L Mid Insula

**Supplementary Figure 1 legend:**

Panels A, B and C display 3 stereotypical seizures originating in the right hippocampus, left basal temporal neocortex quickly evolving in the left hippocampus and left hippocampus without clear left basal temporal involvement at seizure onset. The spatial build-up seen in seizure B as opposed to the broad onset seen in seizures A and C, is suggestive of a left neocortical seizure onset zone, spreading to the right or left hippocampus, though independent left and right temporal foci cannot be ruled out. In panel (D), showing a typical seizure of patient 2, clinical onset (marked) preceded electrographic seizure in depth electrodes, which did not cover the seizure onset zone.

**Supplementary Figure 2**

**
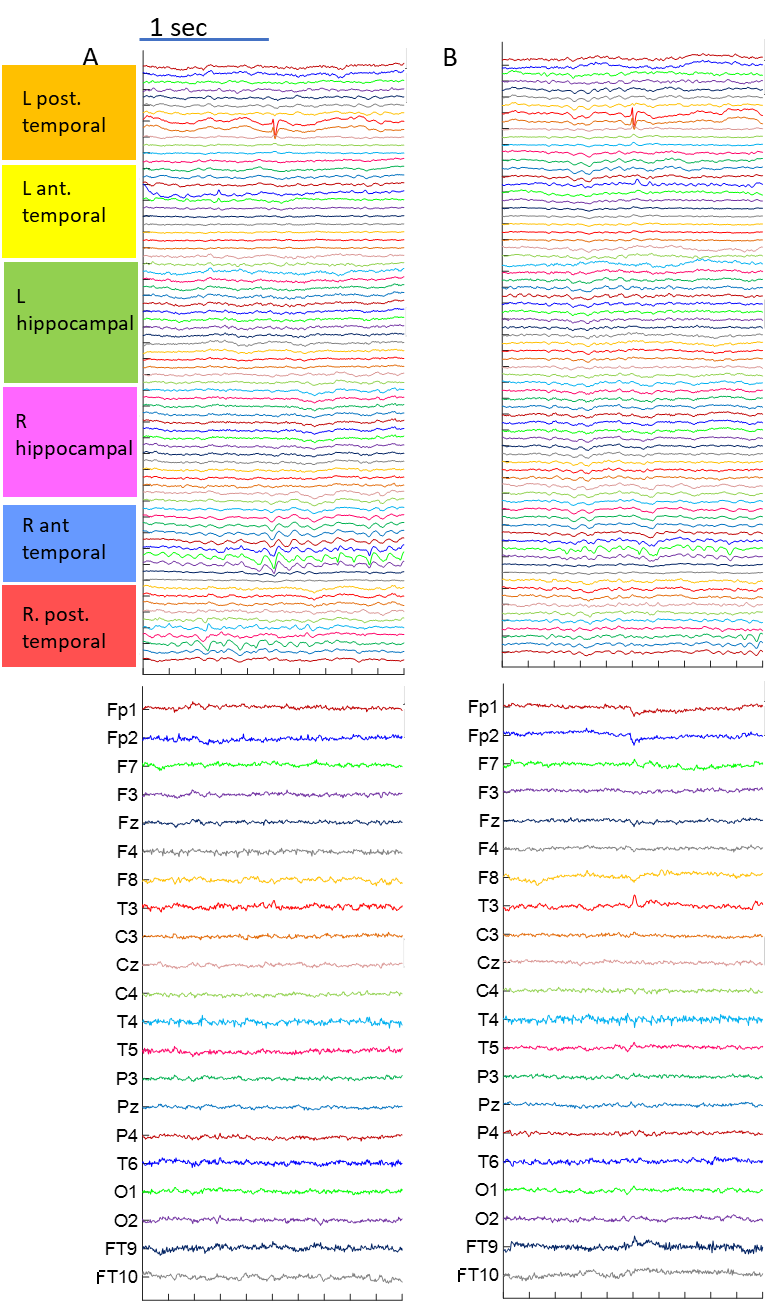
**

**
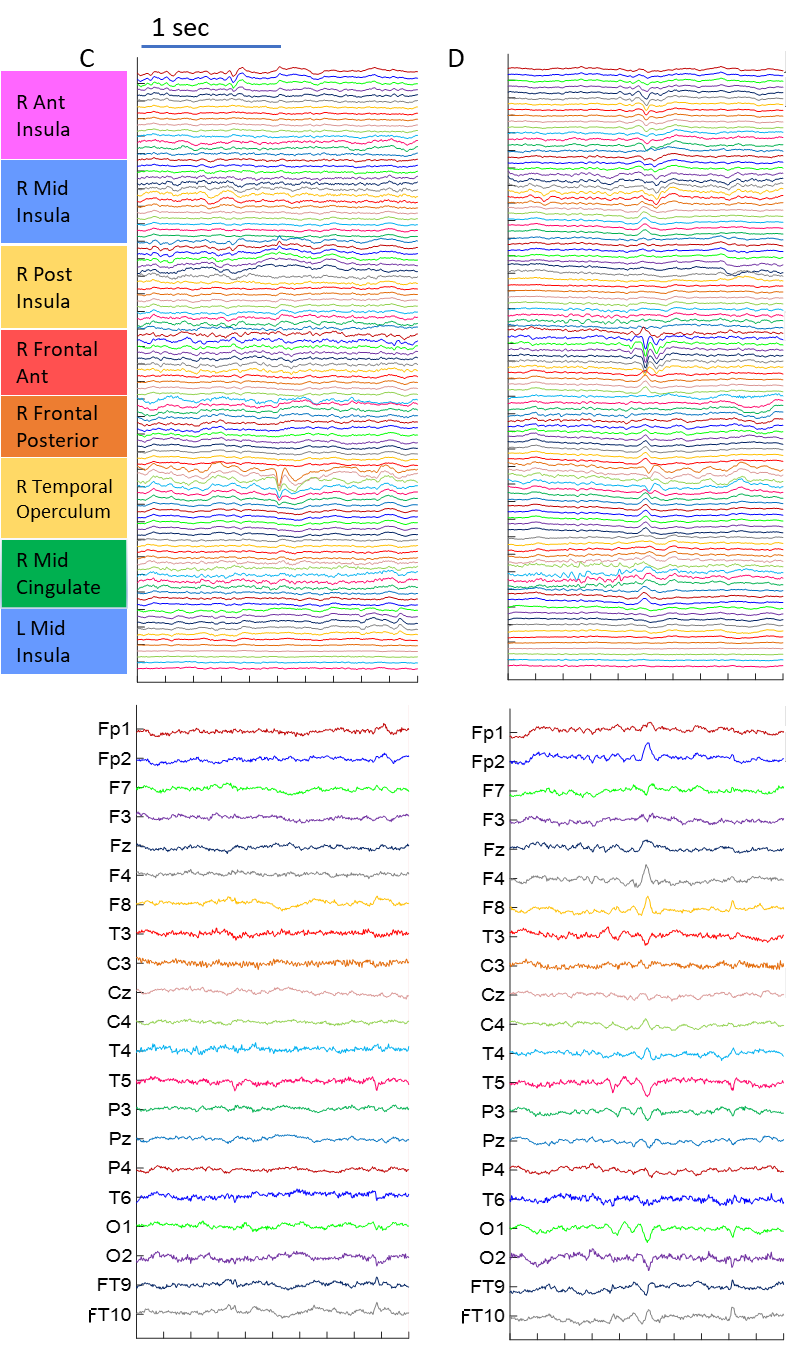
**

**Supplementary Figure 2 legend:**

Exemplary iIEDs with and without scalp correlates: A left posterior temporal spike with (B) and without (A) scalp correlate in patient 1, a right opercular spike without a scalp correlate in patient 2 (C), and a right frontal spike with scalp correlate in patient 2 (D).

**Supplementary video legends**

Videos 1a, 1b and 1c depict the temporal evolution of patient 1's 3 clusters, and videos 2a-d 1c depict the temporal evolution of patient 1's 4 clusters, as described in depth in the main text.

**Bibliography**

[1] F. Tadel, S. Baillet, J. C. Mosher, D. Pantazis, and R. M. Leahy, "Brainstorm: a user-friendly application for MEG/EEG analysis," (in eng), *Comput Intell Neurosci,* vol. 2011, p. 879716, 2011, doi: 10.1155/2011/879716.

[2] R. D. Pascual-Marqui, "Standardized low-resolution brain electromagnetic tomography (sLORETA): technical details," (in eng), *Methods Find Exp Clin Pharmacol,* vol. 24 Suppl D, pp. 5-12, 2002.

[3] J. Kybic, M. Clerc, T. Abboud, O. Faugeras, R. Keriven, and T. Papadopoulo, "A common formalism for the integral formulations of the forward EEG problem," (in eng), *IEEE Trans Med Imaging,* vol. 24, no. 1, pp. 12-28, Jan 2005, doi: 10.1109/tmi.2004.837363.

[4] A. Gramfort, T. Papadopoulo, E. Olivi, and M. Clerc, "OpenMEEG: opensource software for quasistatic bioelectromagnetics," (in eng), *Biomed Eng Online,* vol. 9, p. 45, Sep 06 2010, doi: 10.1186/1475-925X-9-45.

[5] G. Wang, G. Worrell, L. Yang, C. Wilke, and B. He, "Interictal spike analysis of high-density EEG in patients with partial epilepsy," (in eng), *Clin Neurophysiol,* vol. 122, no. 6, pp. 1098-105, Jun 2011, doi: 10.1016/j.clinph.2010.10.043.

[6] C. Plummer, S. J. Vogrin, W. P. Woods, M. A. Murphy, M. J. Cook, and D. T. J. Liley, "Interictal and ictal source localization for epilepsy surgery using high-density EEG with MEG: a prospective long-term study," (in eng), *Brain,* vol. 142, no. 4, pp. 932-951, Apr 01 2019, doi: 10.1093/brain/awz015.

[7] M. Fernandez-Corazza *et al.*, "Source localization of epileptic spikes using Multiple Sparse Priors," (in eng), *Clin Neurophysiol,* vol. 132, no. 2, pp. 586-597, Feb 2021, doi: 10.1016/j.clinph.2020.10.030.

[8] J. W. Meijs, O. W. Weier, M. J. Peters, and A. van Oosterom, "On the numerical accuracy of the boundary element method," (in eng), *IEEE Trans Biomed Eng,* vol. 36, no. 10, pp. 1038-49, Oct 1989, doi: 10.1109/10.40805.

[9] R. S. Desikan *et al.*, "An automated labeling system for subdividing the human cerebral cortex on MRI scans into gyral based regions of interest," (in eng), *Neuroimage,* vol. 31, no. 3, pp. 968-80, Jul 01 2006, doi: 10.1016/j.neuroimage.2006.01.021.
